# Etymologia: Ring Vaccination

**DOI:** 10.3201/eid3002.221909

**Published:** 2024-02

**Authors:** Vijay Sharma, Rajnish Sharma, Balbir B. Singh

**Affiliations:** Guru Angad Dev Veterinary and Animal Sciences University, Punjab, India

**Keywords:** etymologia, ring vaccination, surveillance, containment, vaccination, smallpox, history, viruses

## Ring Vaccination [rɪŋ-væk-sɪ′-neɪ-ʃn]

Ring vaccination (expanding ring, surveillance and containment) is a public health measure designed to prevent spread of disease from infected persons to others. This approach targets persons who have had close contact with confirmed or suspected cases and are at a higher risk of infection by vaccinating them first (Figure).

**Figure:**
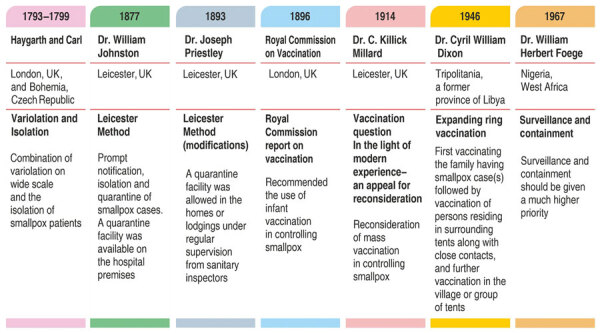
Historical concepts and persons associated with development of ring vaccination strategy.

This strategy has shown remarkable success in combating smallpox caused by respiratory droplet/direct contact–based transmission and shortened incubation for the vaccine. The concept of protecting persons closely exposed to smallpox cases might have its origins in the late 18th century, when the London Small-Pox and Inoculation Hospital was established in 1746. Haygarth (1793) and Carl (1799) suggested systematic variolation of the population and isolation of smallpox cases. In 1877, the Leicester Method, which involved prompt notification, isolation, and quarantine of smallpox cases, was introduced in Leicester (a town in East Midlands, England), and was advocated by local anticompulsory vaccinationists. In 1896, the Royal Commission on Vaccination recommended infant vaccination to control smallpox, although C. Killick Millard, Medical Officer of the Health, appealed for reconsideration. The Leicester method was later supplemented with vaccination or revaccination of contacts in the early 20th century.

After World War II in 1946, despite limited vaccine supplies, Dixon eliminated a smallpox outbreak in the Tripolitania (a former province of Libya) using a method termed expanding ring vaccination. In 1967, Foege and colleagues introduced this concept as surveillance and containment in the smallpox eradication campaign in Nigeria. The strategy proved successful for smallpox because of the disease’s relatively slow spread, mostly through face-to-face contact. The term ring vaccination is now universally used for this process.
